# Early COVID-19 Vaccination of Romanian Medical and Social Personnel

**DOI:** 10.3390/vaccines9101127

**Published:** 2021-10-03

**Authors:** Loredana Sabina Cornelia Manolescu, Corneliu Nicolae Zaharia, Anca Irina Dumitrescu, Irina Prasacu, Mihaela Corina Radu, Adrian Calin Boeru, Liliana Boidache, Irina Nita, Andrei Necsulescu, Razvan Daniel Chivu

**Affiliations:** 1 Department of Microbiology, Parasitology and Virology, Faculty of Midwives and Nursing, “Carol Davila” University of Medicine and Pharmacy, 050474 Bucharest, Romania; liliana.boidache@yahoo.com (L.B.); irina.nita0910@gmail.com (I.N.); 2 Department of Virology, Institute of Virology “Stefan S. Nicolau”, 030304 Bucharest, Romania; corneliu.zaharia@gmail.com; 3 Department of Fundamental Sciences, Faculty of Pharmacy, “Carol Davila” University of Medicine and Pharmacy, 050474 Bucharest, Romania; ancairinadumitrescu@yahoo.com (A.I.D.); irinaprasacu@yahoo.com (I.P.); 4 Department of Birth, Obstetrics and Gynecology Hospital, 100409 Ploieşti, Romania; calinboeru@yahoo.com; 5 Medical Oncology Department, Elias University Emergency Hospital, 011461 Bucharest, Romania; 6 Department of Emergency, Central Military Emergency University Hospital “Dr. Carol Davila”, 010825 Bucharest, Romania; andrei.neck@gmail.com; 7 Department of Public Health and Health Management, Faculty of Midwives and Nursing, “Carol Davila” University of Medicine and Pharmacy, 050474 Bucharest, Romania; razvan.chivu@umfcd.ro

**Keywords:** COVID-19 vaccines, medical and social staff vaccination, mRNA vaccine

## Abstract

Background: In December 2020, the first doses of COVID-19 vaccines arrived in Romania and were made available to medical and social staff. Vaccine hesitancy appeared as a barrier to effectively ending the pandemic. The opinions of medical and social staff influence the opinion of the general population. This study assesess the attitudes, knowledge, and opinion of medical and social personnel toward COVID-19 vaccines and vaccination and the influencing factors. Methods: 1025 persons participated in an online cross-sectional study from March until July 2021. Results: Out of 1021 eligible responders, 719 (70.42%) had been vaccinated: 227 with one dose (22.23%) and 492 with two doses (48.18%). There were 302 responders who were not vaccinated at all. Out of them, 188 refused vaccinations. The participants showed a good understanding and knowledge of SARScoV-2 transmission and treatment. Geographic area, medical profession, and medical experience influenced COVID-19 vaccination (*p* < 0.001). There were no associations between willingness to vaccinate and vaccine/virus knowledge. Most of the responders who were vaccinated or wanted to be vaccinated indicated an mRNA vaccine as their first choice. The variables that were significantly associated with reporting COVID-19 vaccine acceptance after logistic regression were: living in an urban area (Ora = 1.58, 95% CI: 0.98–2.56), being female (Ora = 1.59; 95% CI:1.03–2.44), and being a medical doctor (Ora = 3.40; 95% CI: 1.84–6.26). Conclusions: These findings show that vaccine hesitancy persists in medical and social personnel in Romania, and, hence, it may be reflected in the hesitancy of the general population toward vaccination.

## 1. Introduction

COVID-19 pandemic outbreak officially started on 11 March 2020, according to the World Health Organization [1]. Since then, the lives of people around the world have been dramatically altered [2]. At present, newly identified SARS-CoV2 variants have caused a relapse of confirmed cases worldwide, posing again a serious threat to public health [3].

As of December 2020, vaccine candidates started to be approved, and vaccination campaigns started to take place in all countries over the world [4]. When the first doses of COVID-19 vaccines arrived in Romania, the first categories to which the vaccine was available were medical and social staff. In Romania, the first case of COVID-19 was documented on 26 February 2020 in Bucharest, and the first person vaccinated against COVID-19, on 27 December 2020, was a nurse, involved from the beginning of the pandemic, in the treatment of COVID-19 patients at the National Institute of Infectious Diseases “Matei Bals” from Bucharest.

As vaccination against COVID-19 remains the most effective weapon to stopping the current pandemic, we assessed the opinion, attitudes, and knowledge of Romanian health professionals regarding COVID-19 vaccines and vaccination. We also evaluated the factors that influenced vaccination. In this regard, we supplied a questionnaire to 1025 medical and social professionals and medical students from several Romanian hospitals that were designated as COVID-19 hospitals. We chose this target population because health-related personnel were the first population vaccinated in Romania and the one that is most likely at risk to develop the disease despite the vaccines. The need for vaccination in this category has been well documented and recognized in previous studies [5,6,7] because this population has suffered from mental health issues during the pandemic. Furthermore, health personnel vaccination sets an example for the general population and may influence people’s willingness to vaccinate and increase the vaccination rate.

## 2. Materials and Methods

### 2.1. Study Design, Sites, and Participants

An analytical cross-sectional study was conducted in Romania in six COVID-19 hospitals and two major universities. The hospitals were in the southern part of the country in the following cities: Bucharest, Ploiesti, Craiova, Campina, Buzau, and Medgidia. The universities were “Carol Davila” University of Medicine and Pharmacy in Bucharest, one of the largest universities in Romania, and the University of Medicine and Pharmacy in Craiova. It was addressed to medical staff, health care workers (medical doctors, nurses, midwives, medical students, paramedics), and social staff (social workers) from the main COVID-19 hospitals. The medical students who took part in the study were from the final third year of the faculty, the students who perform clinical internships and are in contact with COVID-19 patients.

### 2.2. Survey Questionnaire and Data Collection

Data were collected with the use of a self-administered questionnaire. The questionnaire was administered online from March to July 2021, after healthcare workers and medical students had access to vaccination and could have completed the vaccination course. In Romania, this period was a lock-down period, a very stressful and complicated period. 

The questionnaire (which can be found in Supplementary Material S1) was composed of 28 questions (some of which were yes/no/I don’t know questions, while others were multiple choice questions) and was distributed by the authors of this publication through media messages in institutional communication groups during the pandemic in the form of a link after it was issued in Google Forms. Informed consent was required and obtained from each willing participant after he or she was informed of the objectives and activities of this study. The questionnaire was able to be submitted only after answering all the questions. For the feasibility of the results, we decided to exclude the sections from the hospital where the response rate was below 80% of the staff employed. 

### 2.3. Ethical Approval

The questionnaire was peer-validated and approved by the Ethics Committee of the Obstetrics and Gynecology Hospital, Ploiesti, Romania (14325/21.12.2020); all the procedures of the study respect the ethical standards of the Helsinki Declaration. Informed consent was compulsory.

### 2.4. Statistical Analysis

For statistical analysis, we used the Microsoft Office package Excel and SPSS 23.0 software. For data processing, the COUNTIFS function in Excel was used to filter and sort the initial database. To establish the associations of two factors (correlations), we used the chi-square test. We calculated the *p*-value with the chi-square statistic test. Variables that are associated (*p* < 0.10) in the unadjusted analyses were further adjusted for demographic factors (i.e., age) using stepwise logistic regressions. The level of significance was set to 0.05.

## 3. Results

In total, 1025 individual answers were received. The responses to the questions are summarized in Supplementary Material S2. We had to exclude 4 responders due to incorrect answers regarding their age. 

In the analyzed group remained 1021 responders. They had a median age of 30 (quartile Q1 = 22, Q3 = 43; limits: 18–75); the majority were women (871; 85.31%) and those who worked in the medical field (923; 90.40%). The rest of the responders worked in the social field (98; 9.60%). Of the total, there were: medical doctors (260; 25.47%), nurses (232; 22.72%), social assistants (33; 3.23%), pharmacists (74; 7.25%), midwives (41; 4.02%), medical students (365; 35.75%), and paramedics (16; 1.57%). The characteristics of the respondents are summarized in Table 1. 

Out of 1021 eligible responders, 719 (70.42%) had been vaccinated: 227 (22.23%) with one dose and 492 (48.18%) with two doses; 302 responders were not vaccinated at all. The COVID-19 vaccine used in our study group was an mRNA vaccine, the first vaccine approved in Romania.

Some of the studied variables were significantly associated with COVID-19 vaccination after stepwise selection: studies (ORa = 1.21; 95% CI: 0.92–1.60) and geographic area of residence (ORa = 1.21; 95% CI: 0.84–1.75) (Table 2).

When analyzing the relationship between sexes, previous SARS-CoV-2 infection, and COVID-19 vaccination, we found no statistical significance correlation; all values were *p* > 0.05. None of these factors influenced COVID-19 vaccinations. The geographic area of residence and university and post-university studies influenced COVID-19 vaccination success. The responders from urban areas were vaccinated at a higher rate than those from rural areas: 614 (85.39%) vs. 105 (14.60%). The responders with university and post-university studies (411, 57.16%) were vaccinated at a higher rate than those without university studies (308, 42.83%).

Another factor that influenced COVID-19 vaccination was experience in the work domain. There was a strong association of years of experience and the number of responders vaccinated; seniority in the work domain was highly associated with vaccination (*p* < 0.001).

In the group of 1021 responders, a small percentage (27.23%, 278) of responders had COVID-19 and were cured. More than half of them, 169 responders, decided to get vaccinated against COVID-19. Having the COVID-19 disease did not influence the willingness to vaccinate.

Being in the medical profession influenced COVID-19 vaccination (*p* < 0.001): medical doctors vaccinated at a higher percentage (88.46%, 230) followed by midwives (68.29%, 28) and pharmacists (63.51%, 47). Surprisingly, medical students chose to vaccinate at a high percentage, close to the medical doctors (70.41%, 257), compared with nurses (56.03%, 130), social workers (57.57%, 19), and paramedics (50%, 8).

As expected, after completing their COVID-19 vaccination, some responders experienced adverse effects. When discussing the correlation between the adverse effects of COVID-19 vaccination and sex, we discovered that women presented more adverse reactions to COVID-19 vaccines than men. The difference was statistically significant: 305 (87.89%) women vs. 42 (12.10%) men out of the 347 responders that presented adverse reactions (*p* = 0.037).

Because the questionnaire was distributed in the early days of the vaccination campaign in Romania and some participants had not had the opportunity to be immunized, while some were immunized with only one COVID-19 vaccine dose (227), we addressed the question, “If you have not been vaccinated yet or have not completed the vaccination scheme, would you like to be vaccinated later when possible?”. Out of 302 (29.58%) respondents that were not vaccinated at all, the majority (188, 62.25%) refused vaccination. All 227 responders who were vaccinated with only one dose wanted to complete that vaccination. The profiles of the respondents who have refused and accepted vaccination are shown in Table 3. 

Of the 188 responders who refused vaccination, the majority (118) believed that COVID-19 vaccine was far too new and more studies were needed to validate it; 43 responders were afraid of adverse reactions, 42 believed that the mRNA contained in COVID-19 vaccine interacts with human DNA, and 24 responders believed that is more useful to get antibodies through getting the disease. There are 37 responders who did not agree with vaccination in general; these represented just a small fraction of 3.62% of the total number of 1021 responders.

Of these 188 responders, nurses (79, 42.02%) and medical students (62, 32.98%) refused vaccination at the highest percentage compared with other categories such as medical doctors, pharmacists, and midwives.

Some variables were significantly associated with COVID-19 vaccine acceptance after stepwise selection, including occupation, sex, residence, and COVID-19 infection (Table 4).

The variables that were significantly associated with reporting COVID-19 vaccine acceptance after logistic regression were living in the urban area (ORa = 1.58, 95% CI: 0.98–2.56), being female (ORa = 1.59; 95% CI:1.03–2.44), and being a medical doctor (ORa = 3.40; 95% CI: 1.84–6.26) (Table 3). The fact that they were or were not infected with SARS-CoV2 was the least important factor that leads to the desire to vaccinate (*p* = 0.436).

When the responders were asked if they knew the routes of SARS CoV-2 transmission, the symptoms of COVID-19, and the treatment applied so far, most of them answered correctly, the majority recognizing all ways of transmission and all the symptoms and treatments applied in hospitals up to the questionnaire date. The responders also correctly acknowledged the means of prevention, and 880 (85.9%) of the 1021 responders indicated vaccination as the best method of prevention.

More than 95% of the responders indicated that this pandemic triggered effects such as economic, sanitary, and educational crises, restriction of rights and freedoms, and online teaching.

When the responders were questioned about the existing COVID-19 vaccines, 892 (87.37%) correctly identified all types of existing vaccines; however, there were responders that knew about the existence of only two (23, 2.25%) or three (105, 10.28%) types of COVID vaccines. We found out that the best-informed responders from our group were medical doctors and students.

Responders were further asked which type of vaccine they preferred, and the majority (about 77.08%, 787 responders out of 1021) indicated an mRNA vaccine as their first choice. However, there were 46 responders that would agree to be immunized with any type of vaccine.

When the responders were questioned about the COVID-19 vaccines and ways of administering them, about half of them (582 responders out of 1021, 57%) answered that two doses are needed. The rest knew that there were vaccines that needed one dose and others that needed two doses.

The responders also correctly identified the contraindications of the COVID-19 vaccines and acknowledged the fact that vaccination is voluntary and a certificate of vaccination is issued after vaccination.

The last question addressed to the responders evaluated the fear of getting COVID-19. We found no association with geographic area (*p* = 0.91), but there was a strong association with the willingness to vaccinate (*p* < 0.001), thus indicating that fear of COVID-19 influences the willingness to get the COVID-19 vaccine.

## 4. Discussion

Recent studies have suggested that the availability of vaccines for medical staff is between 60% and 90% among doctors in Greece (February 2020) and France (March–July 2020) [8,9] and between 40% and 60% among nurses in Hong Kong, China (February–March 2020), and France [9,10].

The Romanian COVID-19 vaccination program started at the same time as other vaccination programs around the world, but vaccine hesitancy appeared as a barrier to effectively ending the pandemic. The importance of opinions, attitudes, and knowledge of Romanian health professionals regarding COVID-19 vaccines and vaccination has fueled the success of the Romanian vaccination campaign. To date, according to the European Center for Disease Prevention and Control COVID-19 Vaccine Tracker in Romania, cumulative uptake of at least one vaccine dose in the total population of Romania onAugust 18, 2021 is only 26.6%, a low value compared with EU/EAA data, which are 62.3% [11].

All around the world, many factors influence the willingness to vaccinate, including safety, efficacy, and side effects [6]. In our study, we found that women presented, more often, adverse reactions to COVID-19 vaccines than men (*p* = 0.037, *p* < 0.05), but despite that, they were more willing to accept vaccination (ORa = 1.59; 95% CI: 1.03–2.44). It is known that there are more women nurses than men.

The opinion of medical and social staff is highly important as they may influence their recommendations and, thus, the opinion of the general population, resulting in an increase in the success of the campaign. The vaccination of medical and social staff in hospitals or other public facilities with large numbers of patients, such as oncology or infectious diseases, helps prevent the spread of COVID-19 among immune-compromised patients [12,13,14,15,16]. However, health workers are not a homogeneous group, and most are not experts in vaccination [17]. Additionally, immunization has not been an important part of their initial training [18]. We found that medical doctors were the first category of health personnel who accepted vaccination (ORa = 3.40; 95% CI: 1.84–6.26).

Our study investigated the opinions, attitudes, and knowledge of Romanian health and social professionals regarding COVID-19 vaccines and vaccination, along with the factors that influence COVID-19 vaccination in a group of 1021 respondents, mainly from southern Romania. While we analyzed a limited sample size, these data may be considered important as they cover medical and social professionals. The respondents were medical and social professionals involved in caring for COVID-19 patients in special COVID-19 hospitals in southern Romania who were directly interested in COVID-19 vaccines and the vaccination process.

The authors are aware that a limit of the representativeness of the study sample is the limitation of the selection of respondents with digital skills, a selection by default, as the studied sample contains only people who can use the internet and electronic documents. This is also suggested by the age of the respondents, with a median age of 30 and the format of the questionnaire, a Google Forms questionnaire, accessible by link and electronically self-administered.

Another limitation of this study is that it analyzed the attitudes of medical staff toward vaccinating against COVID-19 at a specific time during the COVID-19 pandemic, from the beginning of March 2021 to the end of July 2021. It is possible that attitudes towards vaccination may change over time, as major news about vaccines appear daily and political circumstances and epidemiological data change; therefore, longitudinal monitoring is indicated.

One main conclusion of our study is that although most of our respondents had good knowledge about the COVID-19, vaccines, and the vaccination process, not all medical categories were willing to be vaccinated. Unlike previous international studies that showed medical doctors exhibiting favorable attitudes toward COVID-19 vaccines [19], in our study, there were other categories that also agreed with COVID-19 vaccination, such as midwives and pharmacists. According to prior studies developed in Romania, medical students were, in a high percentage, pro-vaccination (88.5% from a study group of 1581 responders) [20]. In our study, we found a slightly smaller percentage than in the previous Romanian study, around 70.41% of medical students from the 365 students who took part in the questionnaire. The categories that supported fewer COVID-19 vaccinations were nurses (56.03%, 130), social workers (57.57%, 19), and paramedics (50%, 8).

Our study showed the existence of a small fraction of 3.62% (37 health professionals out of 1021) who do not agree with vaccination in general. However, all together, in the study group, 188 responders refused COVID-19 vaccination mainly due to its novelty as an mRNA vaccine and poor understanding of disease pathology (the responders believed that the mRNA contained in the COVID-19 vaccine interacts with human DNA and that it is more useful to get antibodies through getting the disease). There were also fears of the side effects generated by the vaccine. Out of these 188 responders, the majority (76, 40.42%) finished college and had a university degree.

Another part of the study focused on the factors that may influence the vaccination campaign. Years of experience in the medical domain was an important factor that supported the acceptance of COVID-19 vaccination (*p* < 0.001), along with the area of residence (*p* < 0.001). This finding supports the previous conclusion that educational programs should be initiated and supported by experienced medical doctors.

This finding indicates the need for continuous medical education or specific educational programs regarding COVID-19 vaccination. We believe our study will generate a correct opinion about COVID-19 vaccines and vaccination and will improve, on the other hand, counseling to all categories of patients and prevent a failure of the current vaccination campaign.

Hesitation regarding the administration of a vaccine among nurses has been demonstrated in numerous studies of influenza vaccination attitudes [21,22,23]. It should not be ignored by public health authorities, given that nurses frequently interact with patients and are in charge of the direct administration of vaccines.

In Romania, there has been a prior negative experience of introducing new vaccines, as was the case with the HPV vaccine’s introduction in 2007, a campaign that ended in failure in 2014 due to a very low acceptance rate in the general population [24].

As the vaccination race continues [25] worldwide, Romanian professionals in the sanitary and social fields will form an opinion on COVID-19 and its prophylaxis through vaccination and may support this campaign while decreasing the number of those who reject vaccination.

## 5. Conclusions

Our findings, which represent data collected right after the start of the national vaccination program, show that vaccine hesitancy persists in medical and social personnel in Romania; hence, it may be reflected in the hesitancy toward vaccination of the general population, which could benefit from specific, targeted public health campaigns.

We believe the key to a COVID-19 successful campaign is first convincing medical and social health staff of the importance of COVID-19 vaccination through educational programs.

The hesitation about a new coronavirus vaccine among health care workers should be a warning sign to public health authorities, as it could have negative effects on the public.

## Figures and Tables

**Table 1:** Vaccination/infectious COVID-19 status and socio-demographic and educational characteristics of respondents to the questionnaire.

Characteristics	N = 1021
Vaccinated against COVID-19	719 (70.42%)
Not infected with SARS CoV2	743 (72.77%)
Sex (women)	871 (85.31%)
Age (median, min–max)	30 (18–75)
Geographic area (urban)	861 (84.33)
Family status: married	545 (53.2%)
Domain of activity: medical	923 (90.40%)
Years of experience (median, min–max)	4 (1–52)
Studies	
High school	311 (30.46%)
Post-secondary school	144 (14.10%)
College/University studies	416 (40.74%)
Post-university studies/PhD	150 (14.69%)

**Table 2:** Logistic regression model for COVID-19 vaccination.

	B	E.S.	Wald	*p*	ORa	Lower CI 95%	Upper CI 95%
Sex (female vs. male)	−0.295	0.206	2.056	0.152	0.744	0.497	1.114
Studies (university and more vs. high school and secondary school)	0.196	0.140	1.949	0.163	1.216	0.924	1.601
Geographic area (urban vs. rural)	0.196	0.186	1.104	0.293	1.216	0.844	1.753
Constant	0.852	0.260	10.773	0.001	2.344	1.410	3.899

**Table 3:** Main characteristics of the responders who refused and accepted COVID-19 vaccination.

Characteristic of the Responders Who Refused	N = 188
Not infected with SARSCoV2	127 (67.56%)
Sex (women)	162 (86.17%)
Age (median, min–max)	26 (19–68)
Geographic area (urban)	149 (79.25%)
Family status: married	66 (35.10%)
Family members infected with SARS-CoV2	90 (47.87%)
Domain of activity: medical	153 (81.38%)
Years of experience (median, min–max)	2.5 (1–42)
**Studies**	
High school	51 (27.12%)
Post-secondary school	40 (21.27%)
College/university studies	76 (40.42%)
Post-university studies/PhD	21 (11.17%)
Characteristic of the responders who accepted	**N = 341**
Not infected with SARSCoV2	241 (70.67%)
Sex (women)	296 (86.80%)
Age (median, min–max)	39 (18–66)
Geographic area (urban)	295 (86.51%)
Family status: married	201 (58.94%)
Family members infected with SARS-CoV2	171 (50.14%)
Domain of activity: medical	311 (91.20%)
Years of experience (median, min–max)	12 (0–43)
**Studies**	
High school	49 (14.36%)
Post-secondary school	55 (16.12%)
College/university studies	173 (50.73%)
Post-university studies/PhD	64 (18.76%)

**Table 4:** Logistic regression model for COVID-19 vaccine acceptance.

	B	ES	Wald	*p*	ORa	Lower CI 95%	Upper CI 95%
Occupation (doctors vs. other health workers and students	1.225	0.312	15.444	<0.001	3.404	1.848	6.269
Sex (female vs. male)	0.261	0.277	0.888	0.346	1.298	0.755	2.232
Residence (urban vs. rural)	0.462	0.245	3.558	0.059	1.588	0.982	2.567
SARS-CoV2 infected vs. not infected	−0.157	0.201	0.608	0.436	0.855	0.576	1.268
Constant	−0.131	0.341	0.148	0.701	0.877	0.450	1.712

## Supplementary Materials

The following is available online at https://www.mdpi.com/article/10.3390/vaccines9101127/s1, Supplementary Material S1: vaccination questionnaire; Supplementary Material S2: questionnaire response.
