# Supplementary material for: Early COVID-19 Vaccination of Romanian Medical and Social Personnel
Source: Vaccines (Basel). 2021 Oct 3;9(10):1127. doi: 10.3390/vaccines9101127 (PMC8541077; doi:10.3390/vaccines9101127)
Supplement: Supplementary file 1 [file vaccines-09-01127-s001.zip › vaccines-1372086-supplementary.pdf]

Characteristic	N = 1021	
**Sex**	Feminine	871 (85,31%)
	Masculine	150 (14,69%)
**Age**	30 (IQR: 22, 43)	
**Geographic area**	Rural	160 (15,57%)
	Urban	861 (84,33%)
**Studies**	College/University studies	416 (40,74%)
	General studies	311 (30,46%)
	Post university studies	150 (14,69%)
	Post-secondary school studies	144 (14,10%)
**Family status**	Married	429 (42,02%)
	Divorced	48 (4,7%)
	Unmarried	544 (53,28%)
**Field of activity:**	Medical	923 (90.40%)
	Social	98 (9.60%)
**Profession:**	Doctor/physician	260 (25.47%)
	Nurse	232 (22.72%),
	Social assistants	33 (3.23%);
	Midwife	41 (4.02%)
	Pharmacist	74 (7.25%)
	Medical student	365 (35.75%)

Question	yes	no

Have you been infected with Covid-19?	278 (27,23%)	743 (72,77%)
Have you been immunized with COVID-19 vaccine?	719 (70,42%)	302 (29,58%)
Have you had any side effects from the vaccination?	347 (33,99%)	594 (58,18%)
Do you have family members who are or have been infected with COVID-19?	534 (52,3%)	487 (47,70%)
If you have not been vaccinated yet, or complete the vaccination scheme would you like to do so?	341 (33.4%)	188 (18.41%)

	**Yes**	**I don't know**
Is the vaccination certificate issued after vaccination?	972 (95,2%)	49 (4,8%)

	**Yes**	**No**	**I don't know**
Does the RNA-Messenger contained in the COVID-19 vaccine interact with human DNA?	139 (13.61%)	634 (62,1%)	248 (24.29%)
Can COVID-19 infection be prevented?	943 (92.36%)	25 (2.45%)	53 (5.19%)

How many types of vaccine do you know there are?

One	1 (0,1%)
Two	23 (2,25%)
Three	105 (10,28%)
More	892 (87,37%)

If you have not been vaccinated, the reason is: I do not agree with vaccination 41 (4.01%)

How many doses are required for the Anti-COVID-19 vaccine?

One dose	3 (0.29%)
Two doses	582 (57%)
in some single-dose vaccines in others in two-dose vaccines	436 (42.7%)

What type of vaccine would you like to get vaccinated with? (Keep in mind that the technologies used by manufacturers to produce the vaccine are different)

any vaccine	46 (4,51%)
Johnson&Johnson	16 (1,57%)
Moderna	23 (2,25%)
I don't want to be vaccinated	87 (8,52%)
Pfizer-BioNTech	633 (62%)
Pfizer-BioNTech, Moderna	91 (8,91%)
Pfizer-BioNTech, Moderna, Oxford-Astra-Zeneca, Johnson&Johnson	26 (2,55%)

What do you consider to be the most effective ways to prevent contact with COVID-19?

Vaccination	48 (4.7%)
Vaccination, wearing protective equipment (mask, visor, goggles, gowns, coveralls, cape, boots, etc.), frequent hand washing (minimum 15 seconds), avoiding crowded spaces and keeping social distance, proper disengagement of equipment protection, decontamination	332 (32.51%)
Vaccination, wearing protective equipment (mask, visor, goggles, gowns, coveralls, cape, boots etc.), frequent hand washing (minimum 15 seconds), avoiding crowded spaces and keeping social distance, proper disengagement of equipment protective	108 (10,58%)
